# The role of models in the covid-19 pandemic

**DOI:** 10.1186/s13584-022-00546-5

**Published:** 2022-10-20

**Authors:** David M. Steinberg, Ran D. Balicer, Yoav Benjamini, Hilla De-Leon, Doron Gazit, Hagai Rossman, Eli Sprecher

**Affiliations:** 1 Department of Statistics and Operations Research, Tel Aviv University, Tel Aviv, Israel; 2 Innovation Division, Clalit Health Services, Clalit Research Institute, Tel Aviv, Israel; 3 School of Public Health, Faculty of Health Sciences, Ben Gurion University of the Negev, Be’er Sheva, Israel; 4 Faculty of Biology, Technion-Israel Institute of Technology, Haifa, Israel; 5 Racah Institute of Physics, Hebrew University of Jerusalem, Jerusalem, Israel; 6 Department of Computer Science and Applied Mathematics, Department of Molecular Cell Biology, Weizmann Institute of Science, Rehovot, Israel; 7 Division of Dermatology, Tel Aviv Sourasky Medical Center, Tel Aviv, Israel; 8 Faculty of Medicine, Tel Aviv University, Tel Aviv, Israel

**Keywords:** Forecasting, Nowcasting, SIR Model, Agent-Based models, Data Analysis, Statistics

## Abstract

Mathematical and statistical models have played an important role in the analysis of data from COVID-19. They are important for tracking the progress of the pandemic, for understanding its spread in the population, and perhaps most significantly for forecasting the future course of the pandemic and evaluating potential policy options. This article describes the types of models that were used by research teams in Israel, presents their assumptions and basic elements, and illustrates how they were used, and how they influenced decisions. The article grew out of a “modelists’ dialog” organized by the Israel National Institute for Health Policy Research with participation from some of the leaders in the local modeling effort.

## Introduction

Models play a significant role in all branches of science. They are used to abstract and to represent natural phenomena, and to understand complex systems and processes. Models have been important tools during the SARS-CoV-2 pandemic, guiding efforts to track disease status, to understand its transmission dynamics, to forecast disease levels and health system loads, and to guide public policy. The high profile media coverage of the pandemic, as might be expected, gave unprecedented public exposure to the role of models in science.

In response to the developments described above, the Israel National Institute for Health Policy Research (NIHP) organized a “modelists dialog” in early February 2022. The meeting included presentations from five scientists who have been actively engaged in modeling data on SARS-CoV-2 and two expert discussions. It drew an online audience of more than 250 participants, emphasizing both the importance of and the wide interest in the use of models.

The purpose of this article is to summarize and synthesize the ideas and approaches presented at the meeting, noting both the accomplishments and the challenges. We will not present the models themselves in any detail; readers interested in the precise formulations and results can find them in the papers published by the modeling teams, which we include as references. Many challenges still lie ahead. With further waves of the pandemic an ever-present threat, efforts are needed now to establish the scientific knowledge and the infrastructure that will make modeling increasingly effective for supporting public health policy.

Orly Manor, the director of the Institute, described the background and motivation for the meeting. Nachman Ash, the Director General of the Ministry of Health, noted the importance of models in deciding on policy, both in the ministry and in discussions by the government. The main part of the meeting was the presentation of methods by the five modelists: Hilla De-Leon (Technion), Doron Gazit (Hebrew University of Jerusalem), Amit Huppert (Gertner Institute), Hagai Rossman (Weizmann Institute of Science) and Eli Sprecher (Tel Aviv Sourasky Medical Center, TASMC). All the speakers emphasized that they worked in larger research groups and that the ideas and results reflected the combined effort of the group. The talks were followed by discussions from Yoav Benjamini (Tel Aviv University) and Ran Balicer (Clalit Medical Services and Ben Gurion University and chair of the national cabinet of experts advising the Israeli government).

## The goals of modeling

An essential issue for framing the discussion here is to delineate the goals that the models aim to achieve. Many different aspects were described; they are listed in Table 1.

**Table 1:** Problems that were addressed with the help of mathematical and statistical models

Provide a “reference scenario”.
Track the current status of the pandemic.
Estimate the pandemic’s dynamics.
Generate short-term predictions (“nowcasting”).
Generate long-term predictions.
Determine ahead of time the need for infrastructural changes in healthcare facilities to accommodate predicted influx of severe cases.
Answer specific questions.
Understand the infection-disease-hospitalization-death cycle.
Assess interventions such as lockdowns or vaccination.

All the speakers and discussants emphasized application by decision makers as an important aspect of modeling, with the implied need to focus modeling efforts in directions that address the dilemmas faced by those making decisions.

## Data and models

Another common theme that influenced all the modelists was the need to link their efforts to the available data; the inability to obtain data imposed limitations for many of the modeling teams. The critical role of data availability for modeling was mentioned by several of the speakers and was especially emphasized by Benjamini; it was a substantial part of his discussion.

Table 2 describes some of the important issues that were raised. Many of them are part of the InfoQ framework [1]; others add facets to that framework.

**Table 2:** Issues with data quality and availability

Issue	Description
Resolution	Often data were available only at broad, summary scales. Some models required more detailed, individual information. For example, the daily number of infected was a standard measure for tracking the status of the epidemic, but (i) it was available at the country level and (ii) with no age breakdown for a long period of time. Hence it had only limited information for modeling the effects of social contact.
Integration	Data came from many different sources, including the Ministry of Health, hospitals, the health funds, the Central Bureau of Statistics, Ben Gurion airport, etc. Integrating the data was challenging, especially in early stages of the epidemic. Legal restrictions also limit the ability to combine these data for use in modeling.
Uniformity	The use of data from diverse sources also highlighted the need for uniformity in recording and reporting. For example, hospitals and the Ministry of Health were not always synchronized with regard to defining which patients should be counted as “severely ill” COVID-19 cases.
Quality	Are the data accurate and reliable? When they are combined across different sources or time periods, are they uniform? For example, a change in the definition of what constitutes a “severely ill” COVID-19 patient can have dramatic impact if no adjustment is made in models. Similarly, reported data on infection rates, and fraction of positive tests, are affected by the false positive and negative rates of the testing protocols.
Completeness	What are essential features that are missing from the data? For example, knowledge of day of exposure to the virus is relevant for some of the models, but was generally not provided.
Temporal Relevance	Many models focused on “nowcasting”. Effective use of these models requires rapid data availability. The necessary data were not always immediately obtainable.
Chronology	Time course data was important for many models. For these models, it is essential to know and to correctly model the time lags typical for, say, time from exposure to infection, infection to recovery or infection to hospitalization and to severe illness and death.
Cohort Relevance	Some models involved data borrowed from other settings, for example social contact data from the pre-COVID-19 period, or data on infection rate or disease severity from other countries. Assessments were needed to determine whether these data could safely be used to drive models for Israel.

Data is an essential input to all the modeling efforts. Even with ideal models, prediction accuracy cannot be any better than the quality of the data. Inaccurate or incomplete data inevitably lead to biased predictions. Moreover, often the nature of the data dictated the type of modeling that was possible. For example, some of the infection models required data on daily infection numbers broken down by age groups. These models could not be used early in the pandemic, as data were not available at that level of granularity. Similarly, the efforts by the Ministry of Health were limited, in comparison to the health funds, by lack of individual level data on, for example, comorbidities and general health-conscious behavior.

An article in the Scottish press highlights the problems that result from problematic data. (https://www.heraldscotland.com/news/19932323.public-health-scotland-pulls-covid-case-rate-data-claims-demonstrates-conclusively-vaccines-not-working/) Weekly data from Public Health Scotland (PHS) for the end of January and the beginning of February 2022 showed higher rates of COVID-19 infection among vaccinated than among unvaccinated in Scotland. The reports were quickly exploited by vaccination opponents to support claims that vaccination is driving infection. However, subsequent review by PHS found that the rates were based on inaccurate data on the size of the vaccinated and unvaccinated populations and were convinced that the resulting over-estimation of the number of unvaccinated artificially reduced their infection rate.

## Model elements

The models covered a range of approaches, from regression analysis to stochastic epidemic models. The former are largely empirical models, whereas the latter are “first principle” science-based models. This is a natural basis for distinguishing between models.

There are several advantages to the science-based models.

- They are derived from first principles that mirror scientific understanding of how epidemics spread in a population. One important consequence is that their assumptions are transparent and open to criticism; another is that it is not difficult to introduce modifications that may make them more realistic.
- They have been effectively used in modeling many infectious diseases.
- They have parameters that dictate the dynamics of an epidemic.
- Straightforward modifications allow stratification of the models, say by age groups, although this leads to a substantial increase in the number of parameters.

The major advantage of the empirical models is their simplicity and their focus on the primary task of providing accurate forecasts. The assumptions that drive the science-based models can turn from a strength to an Achilles heel if they prove to be inaccurate. One of the lessons repeatedly learned throughout the COVID-19 pandemic has been the ability of the virus to call into question commonly accepted truths regarding infectious disease spread.

Since the 18th century, when Swiss mathematician and physicist Daniel Bernoulli developed mathematical models to study how variolation could be used to diminish the spread of smallpox, researchers have sought to develop models that can examine and explore the dynamics of infectious disease transmission. In today’s world, and especially during the SARS-CoV-2 pandemic of the last two years, models are the only means of predicting disease spread and thus are essential for national and international decision-making. For further discussion about the need for mathematical models in epidemiology, see [2, 3].

The models can be roughly divided into three categories:

1. Fully empirical models, i.e., regression, machine learning and deep learning. These statistical models are very powerful tools which use known data to predict the future, and can also accommodate large amounts of data. The main drawback to statistical models in COVID-19 is their inability to predict new and future confirmed cases in the presence of changing conditions, for example a scenario of a new variant or the immunization of the population.

However, for predicting the clinical outcome of a COVID-19 patient following infection, statistical models are important, since disease progress depends heavily on the patient’s health status and medical condition.

2. Mathematical models for population disease spread. These models use a set of coupled differential equations to predict the spread of disease. The SIR methodology [4] and its refinements, such as the SEIR model [5], are the outstanding examples of this class. They have been the dominant approach in the scientific literature for studying infectious diseases and were applied by several of the modelists. SEIR stands for “Susceptible, Exposed, Infected, Removed” which serve to decompose the population. The model describes an epidemic via movement of the population from one compartment to another. Individuals who are susceptible become exposed, then become infected, and finally are removed from the population. Removal can be either by cure or by death. Transitions from one state to another are governed by rate equations and resulting sojourn time distributions. The former describe the rate at which individuals move from one compartment to another, the latter the length of time they remain in a state before moving.

The single most important parameter in these models is the reproduction number, *R*, which relates the average number of susceptible individuals that will be infected by a newly infected individual, and can be estimated from data on the population counts in each compartment. The reproduction number became a mainstay of monitoring and reporting throughout the epidemic. From the onset of the pandemic, all Israelis became familiar with the idea that hearing *R > 1* on the evening news was a sign that things were getting worse.

See [6, 7] for more detailed information and a review of applications of the SIR family to modeling and forecasting annual influenza outbreaks.

The modelists who remained at the empirical end of the spectrum emphasized regression techniques. These models link an outcome ${Y_t}$on day *t* to *k* predictor variables ${X_{1,t}},{X_{2,t}}, \ldots ,{X_{k,t}}$that might be relevant for predicting the value of ${Y_t}$. Often the predictors were the outcome itself, or other related variables, recorded on earlier days.

3. Agent-based (or particle) models. These are mathematical models that operate at the level of the individual person rather than the population as a whole. One of the disadvantages of population-level mathematical models is their inability to model system dynamics, in particular when various population subgroups are characterized by different dynamics. In principle, this can be reflected by modeling the population as a sum of the sub-populations, with each one characterized by features unique to it, such as age, dynamics, etc. Since non-pharmeceutical interventions (NPIs) play an essential role in controlling COVID-19, a geography-based model is necessary because these NPIs fluctuate across countries. Also, a successful model should divide the population into several age groups, matching their varying patterns of social interactions. Making the model realistic leads to a large number of groups, though, and would require writing a different equation for each, which complicates the model greatly.

One effective way to overcome these complications is to use instead an agent-based model which represents every single person by a unique “particle”. This leads to a very granular model, but with easy-to-understand rules governing social interaction for each individual, based on the subgroup of the population to which the individual belongs. Hence, microsimulation modeling comes into play in which we have a high degree of heterogeneity, with multiple individuals, each behaving differently. The modeling and simulation proceed by allowing all the individuals to behave and interact according to these rules. Then the resulting macroscopic impact on society is observed.

Other modeling approaches were also used. Some modelists took a translational approach, using science-based models developed with related settings in mind and demonstrating that they could be effectively applied to COVID-19 data. Others were at more of a middle ground on the empirical-mechanistic axis, using process analysis to decompose the route from predictors to outcomes into more detailed steps and then applying empirical analysis to these building blocks.

In this paper, we show how all three types of models have been used in Israel by different research groups to model the spread of the coronavirus under various constraints (NPIs, effective vaccines, etc.) and the clinical course of COVID-19, e.g., predicting the a patient’s health status during the period after infection or hospitalization.

The models in practice.

The SIR/SEIR family was used directly both by Gazit and his partners at the Hebrew University and by Huppert and the team at the Gertner Institute. Both groups used the age-stratified refinement of the model. Gazit’s group also used a model developed by De-Leon and Pederiva [8] which is described below. They used the models to produce accurate predictions of infections, severely ill, and mortality for both the short-term and for periods extending to 5–6 weeks ahead. The team also developed a method for estimating *R* using only very recent data, adding valuable temporal relevance to the estimates. An important contribution of the group to the Israeli cabinet deliberations was their use of the models to assess the effects of policy interventions. In December 2020, taking account of international data relating quarantines and lockdowns to reduced infection and severe illness, they quantified the effect of such restrictions on near-term impact for Israel and compared alternative times for their implementation. Similar analyses were used to predict the effects of the vaccination campaign [9]. These changes highlight the role these measures had in tempering the impact from widespread infection. The omicron variant, which began to dominate infections in December 2021, was both more infectious and less severe than the previous variants. Both of these properties are essential ingredients of good predictions and thus posed new challenges. The delayed onset of the omicron wave in Israel, due to limiting entry to Israel at Ben Gurion Airport, made it possible to adjust the models by incorporating data on omicron from other countries with earlier initiation times. The resulting model-based predictions played a role in the decision to avoid implementing another lockdown in January 2022.

Huppert and the group from Gertner also found that the SIR/SEIR models produced accurate short-term forecasts. Their age-stratified model required as input both stratified infection counts and social contact data for each pair of age groups. The former came from the Ministry of Health, but there was no official source for the latter. The team used Google mobility indices to fill in the gap. After Israel commenced its vaccination campaign in late December 2020, vaccination status was added as a further stratifying variable. The models further assumed that infection counts would follow Poisson distributions about their expected values. This assumption was borne out in the data and gave good fits. The models adapted well to the onset of the omicron variant. By the end of the first week of January, 2022, early in the omicron wave, the model provided accurate forecasts of how the infection counts would increase and when they would peak.

De-Leon and her colleagues at the University of Trento derived an innovative model inspired by physics. This novel approach uses basic principles of statistical physics, in the spirit of Monte Carlo algorithms, to define an “agent-based” model, in which each individual in the population is explicitly represented. This class of models has a rich history; see for example [10, 11]. De-Leon’s model treats individuals as “particles” in a physical system, with the probability of disease transition a function of the distance between the associated particles. Social mobility is reflected in the model by a parameter that governs motion of the particles within the domain of the system. For details, see [8, 12]. Although agent-based models are developed at the “micro” level of individuals, it is common to assess their value by their ability to mimic macro-level behavior. Here this meant comparisons of the model predictions to observed infection patterns. De-Leon reported close tracking to observed Israeli and UK data thru all the early stages of the epidemic. The model also adapted well to the effects of the vaccination campaign and the waning effect of the vaccine after about 5 months [12, 13]. The particles can be divided into many sub-populations, making stratification easy to include [14].

Rossman, Gazit and Sprecher all described efforts to predict the number of COVID-19 patients requiring hospitalization and treatment in intensive care. This was a major concern early in the epidemic, when it appeared that the extent of available respirators would fall well short of the number of patients in need of them. Rossman emphasized, as well, the importance of the models as a basis for comparison and policy evaluation. His group looked at questions like whether the length of hospital stays for COVID-19 patients were decreasing over time and what was the effect of the vaccination campaign at a population level [14]. The model that he and his colleagues developed is a compartment model similar in nature to the SIR model, but focusing on the stages that arise following infection. Does hospitalization occur? If so, how long is the patient in the hospital? How long does it take for patients to move from standard care to intensive care? Initial efforts to work with simple count data were limited in their ability to answer important questions, as they failed to account for the full time course of patients who were currently hospitalized but without final outcomes. Higher resolution data were needed that traced these individual flows. Once those data were obtained, the team resolved the analysis problems using techniques from statistical survival analysis to account for the censoring of patients still hospitalized, Cox proportional hazards regression models to assess factors affecting sojourn times, and competing risk analysis to account for the different outcomes that might lie ahead [15]. Among the interesting findings was that in-hospital death rates for COVID-19 patients were higher during times of heavy load [16].

Gazit’s team reported similar methods and results to those presented by Rossman for the Weizmann group. Gazit also reported on the effective use of sojourn time models and infection counts to forecast the number of patients who would enter the hospital.

Sprecher reported on the modeling efforts at TASMC. The goals were to facilitate planning and preparation in the hospital. In addition to the challenges of managing COVID-19 patients, TASMC was concerned about care for non-COVID patients, due to the resources that had to be diverted from regular services. With the help of an international advisory panel, the TASMC task force produced a dashboard for continual and up-to-date monitoring of patient loads. For planning ahead, the team focused on short-term forecasting (1–2 weeks ahead) of the number of patients, including a breakdown by status with forecasts of severely ill or patients in need of ventilation. The forecasts were computed from regressions that use as input recent infection data, smoothed to remove weekly trends. As more data were accumulated, the model also incorporated information on the typical sojourn time from infection to severe disease. Sprecher noted that one weakness of the model is a tendency to over-predict peak loads. Like Gazit’s group, the TASMC team used data from foreign sources to revise the model for accurate predictions when the omicron variant became dominant. He also noted that the usefulness of the model was assessed in terms of whether the forecasting errors were small enough to permit the hospital to function successfully, a goal that was consistently achieved.

## Models, policy and decision making

Did the models influence policy and decision making? Ash gave a positive answer in his opening remarks, as did Balicer in his discussion. At the meeting, the modelists noted several areas where their work was instrumental in decision making, but generally focused more on the details and results of the models than on their impact. For example, Gazit pointed to the influence of the models on decisions to limit mobility and the number of participants in large indoor events. Subsequently he added some of the direct links to policy described earlier. Huppert noted the value of the models from the Gertner Institute in establishing initial reference scenarios. Sprecher spoke as both a modelist and a decision maker and it was clear that the work by his task force directly affected policy at TASMC.

Effective communication is essential to link decision making to modeling goals and results. Balicer emphasized that modelists need to be aware of the different needs of decision makers as the epidemic progressed. Their initial concern was to have reasonable scenarios that could provide broad limits for possible action. This was soon followed by accurate nowcasting and eventually advancing to the ability to assess in advance the potential effects of interventions. Benjamini pointed to the variety of modeling groups in Israel working on COVID-19 data, speculating that perhaps this variety, rather than leading to a unified and harmonious voice, created a cacophony that detracted from the ability to influence national leaders.

Benjamini stressed the need to describe uncertainty well. That is a challenging task, as there are multiple factors that contribute to variability. These include limitations and inaccuracies in the model, and the capriciousness of the data on which they are fitted. The well-known quote from George Box that “all models are wrong, but some are useful” was mentioned by several speakers. Benjamini’s call echoes that of an international collection of experts who published a “model manifesto” urging modelists to recognize shortcomings and to accurately portray resulting uncertainty [17]. Balicer also emphasized the need to recognize that the models will always have some failings, especially for purposes of long-term predictions.

In quantifying uncertainty for model predictions, Gazit stressed that the uncertainty regarding behavioral inputs likely dwarfed uncertainty as to the exact form of the model; ergo many alternative models can still be useful. In particular for evaluating potential policy options, he noted that they depend heavily on assumptions about how public behavior will change. For example, will people comply with a lockdown? Will they agree to get a vaccination? Gazit argued that this level of input uncertainty can best be described by considering a range of possible response patterns, from optimistic to pessimistic, leading to a corresponding range of predictions. That allowed him and his colleagues to highlight the uncertainty in the predictions, but at the same time to attach numerical values to those scenarios; he found that hard numerical predictions were essential input for those making national policy.

Balicer and Benjamini also mentioned the importance of accurate communication to the public. Reflecting uncertainty there is crucial, as the media often want a single answer and resist the idea that model results and predictions are subject to variability.

## International perspective

The use of mathematical models and the challenge of obtaining timely, accurate and clear data were by no means unique to Israel. Rosenfeld and Tibshirani [18] summarized articles in a special collection devoted to monitoring and forecasting the COVID-19 pandemic, with emphasis on application in the United States. They state early in their article that “we ended up shifting our focus to nearly entirely on the data end of the spectrum”. The forecasting teams in the US faced all the challenges listed above, with the additional burden of having to work with much more diffuse, and less uniform, reporting than in Israel. One of their initial headers is “Understanding the data generation process is critical for downstream applications”. With regard to timeliness, Rosenfeld and Tibshirani [18] noted that even though provisional data often are not complete, good historical records on such data can still make them very valuable for modeling and prediction.

In Italy, the lack of data prompted a petition, signed by 1400 statisticians and researchers, urging the government to make data available for analysis. The same issue was noted as a significant area for improvement in Australia by Trewin and Fisher [19].

The Royal Statistical Society (RSS) formulated a 10-point proposal for improving the ability to understand and respond to a pandemic [20]. Data-related issues resonate throughout the proposal. Here are some specifics. At the top of their list the RSS stressed the need to “invest in public data”. Specifically they called for a review of the current situation, including systems and structures for data collection, levels of investment, assembling data from diverse sources, both within the United Kingdom and at an international level, and better coordination between the data infrastructure and the ensuing data analysis. The second point emphasized the importance of making data, and the analysis based on it, openly available to ensure public trust. This idea recurs in the third point, leading to a recommendation to invest in a central portal for collecting official public health data, operating “under Open Data principles”, and to designate a framework for publishing results from the data. Point 7 notes that “paucity of data” hindered initial efforts to respond to COVID-19 and calls for an effective ongoing surveillance program to monitor epidemics and inform decision-makers.

Molenberghs [21] described the response to COVID-19 in Belgium, presenting a broad picture of biostatistical involvement, much of it centered on data and modeling. In particular, he highlighted the importance of modeling in assessing the likely effect of NPIs. Mathematical models played a key role in the Belgian decision to maintain more stringent NPIs over Christmas and New Year of 2020–2021. The models showed potential for a high peak; the continued restrictions helped to keep COVID under control. Official data in Belgium were spread over several different administrative tiers; this led to difficulty in establishing a unified national database with the information needed for all the modeling efforts. The need to adjust data for underreporting added challenges, both in modeling the data and in presenting results, analyses and predictions to the public [21, 22].

Germany also was faced with a need to produce effective summaries and nowcasts from limited data, in particular due to delays in the reporting system for individuals who tested positive for COVID-19 [23]. Nonetheless, reporting raw, unadjusted infection rates was common. This practice drew sharp criticism from Fritz et al. [23] Instead, they stressed the need for “nuanced data analysis”; in short, for summaries that use statistical models to adjust for the reporting mechanism and produce more accurate nowcasts of infection.

Dattner et al. [24] provided additional comparisons to what happened in other countries. Di Serio et al. [25] also described challenges for understanding COVID-19 that arose from lack of proper data for modeling and analysis.

Ioannidis, Cripps and Tanner [26] presented a critical summary of the modeling and forecasting efforts in the United States. Although published in 2022, it is largely based on a blog that went public in June 2020 and the results emphasize the poor performance of forecasts made during the first months of the pandemic, when good data were scarce and scientists and modelers were making adjustments to improve the match of their predictions to observed outcomes. Data problems (and a host of others) were cited as a key reason for the inaccuracy of the forecasts. The authors conclude by emphasizing the need for predictions to be accompanied by an accurate description of uncertainty.

The challenges of developing data-based policy led to broad international contacts, including sharing data and the insights gained from analyzing and modeling them. The organized data collection mechanisms and the rapid rollout of the initial vaccination in Israel prompted many countries to seek support. The authors specifically noted several countries that approached Israel for data and analyses: the United States, Switzerland, Belgium, France, the United Kingdom, Japan, Germany and Poland.

## Looking ahead

We noted early in the article the close interplay between models and data. Several ideas came up in the talks and discussion about how to build better data infrastructure to support such modeling efforts in future epidemics. For example, Balicer called for the creation of a central, national health information database. Benjamini pointed to the need for data at relevant spatial and temporal resolutions; for example, hospital forecasts would be better served by infection data in their catchment areas than by national data. Rossman noted the need for patient-specific data for the models his group developed and the importance of fusing different data sources in an appropriate manner [27] - these may include digital surveillance tools such as the symptoms survey employed in Israel during the first wave [28] and other emerging population-wide technologies such as wastewater surveillance. Huppert and Balicer commented on the difficulty to predict how people will respond to interventions that affect their social and economic freedom, and the associated need for data and data collection methodology on social mobility and contact, key inputs to the age-stratified models.

There was full consensus among the speakers and discussants that modeling must be an essential tool in guiding public response to an event like the COVID-19 pandemic. Models are necessary to track the pandemic, to assess policy options and to guide decision makers. There was also agreement that, to be useful, modeling efforts need to be directed to the questions that are central to policy.

There was not a consensus as to which models best served those purposes, or on how to focus the attention of national leaders on those models, their conclusions and their recommendations. One of the initiatives that came up was the need for an open and critical forum for discussion and interchange among modelists and other informed scientists. The goal of such dialog should be to critically examine the underpinnings of the models, their results and their predictions. Ideally, this could lead to scientific consensus and the unified and harmonious voice sought by Benjamini. Clearly, the dialog would need to be open and frank, with critics free to express their reservations about models. Huppert added to this the need for better communication between the modelists and the decision makers.

Rosenfeld and Tibshirani [18] added an interesting complementary thought in their summary. They pointed to failures of the forecasts in the US as (i) evidence that there are still many details of epidemic spread that we don’t understand; but (ii) the data from the COVID-19 epidemic is thus an ideal laboratory in which to refine and improve our knowledge. They see this as essential to making the world better prepared to deal with future epidemics. Their second point emphasizes the value in continued curation and quality improvement of the Israeli COVID-19 data and the need to make it available as a resource for research in modeling.

The proliferation of COVID-19 modeling groups in Israel was a spontaneous grassroots development. It included many scientists, all working pro bono, and interested in contributing to the national response. It is important to have a diversity of approaches and opinions on how to most effectively use modeling to guide policy. However, this diversity also runs the risk of creating confusion and controversy. Moreover, it may have the dangerous consequence of politicizing the process, with leaders choosing the experts whose recommendations support their prior opinions. These risks are especially problematic in the initial stages of an epidemic, when reliable scientific advice is crucial, and the need for rapid response rules out the broad and open discussion that typifies science. Rosenfeld and Tibshirani [18], and Ioannidis et al. [26], described a similar situation in the United States, with multiple groups doing modeling. We suspect that the presence of parallel modelers was common to most countries, but we do not have any hard evidence to that effect.

Israel needs to be better prepared for future epidemics. That will require developing infrastructure and expertise within the Ministry of Health and, in particular, the Management Team of Epidemics, and establishing effective communication channels between the experts and the policy leaders. In a crisis such as COVID-19, it will often be necessary to bring additional experts into the modeling and advisory loop. Professional organizations in relevant domains might be useful to help organize such efforts. The National Academy of Sciences can play an important role; the objectivity and inter-disciplinary breadth of the Academy give it a unique status that could be leveraged in coordinating the efforts of all the academic and industry experts who wanted to contribute. To do so requires action now to set up the necessary organizational framework; we cannot afford to wait for the next crisis.

## Data Availability

Not relevant for this paper.

## Abbreviations

NIHP: National Institute for Health Policy.
NPI: nonpharmacological intervention.
PHS: Public Health Scotland.
SARS: Severe Acute Respiratory Syndrome.
SEIR: Susceptible, Exposed, Infected, Recovered.
SIR: Susceptible, Infected, Recovered.
TASMC: Tel Aviv Sourasky Medical Center.
UK: United Kingdom.
US: United States.
